# Expression of epidermal growth factor receptor is an independent prognostic factor for esophageal squamous cell carcinoma

**DOI:** 10.1186/1477-7819-11-278

**Published:** 2013-10-16

**Authors:** Qifeng Wang, Hongxia Zhu, Zefen Xiao, Wencheng Zhang, Xiao Liu, Xun Zhang, Jie He, Kelin Sun, Lvhua Wang, Ningzhi Xu

**Affiliations:** 1 Department of Radiation Oncology, Cancer Institute and Hospital, Chinese Academy of Medical Sciences (CAMS) and Peking Union Medical College (PUMC), No.17, Nanli, Panjiayuan, Beijing 100021, People’s Republic of China; 2 Laboratory of Cellular and Molecular Biology, Cancer Institute and Hospital, Chinese Academy of Medical Sciences (CAMS) and Peking Union Medical College (PUMC), No.17, Nanli, Panjiayuan, Beijing 100021, People’s Republic of China; 3 Department of Pathology, Cancer Institute and Hospital, Chinese Academy of Medical Sciences (CAMS) and Peking Union Medical College (PUMC), No.17, Nanli, Panjiayuan, Beijing 100021, People’s Republic of China; 4 Department of Thoracic Surgery, Cancer Institute and Hospital, Chinese Academy of Medical Sciences (CAMS) and Peking Union Medical College (PUMC), No.17, Nanli, Panjiayuan, Beijing 100021, People’s Republic of China; 5 Present working address: Department of Radiation Oncology, Sichuan Cancer Hospital, No.55, Fourth Section, Renmin South Road, Chengdu 610041, People’s Republic of China

**Keywords:** Esophageal cancer, Esophageal surgery, Operations, Statistics, Survival analysis, Tumor markers, Fiducial markers

## Abstract

**Background:**

The overall survival of patients with esophageal squamous cell carcinoma (ESCC) remains poor. Prognostic predictions in ESCC are usually based on histological assessment of tumor invasion and lymph node metastasis, but a biomarker with better predictive accuracy could be more useful. Because overexpression of epidermal growth factor receptor (EGFR) has been associated with poor prognosis, this study investigated whether EGFR is an independent prognostic factor for overall survival and disease-free survival of ESCC patients.

**Methods:**

ESCC tissue specimens from 243 patients obtained during surgical resection between 1980 and 1997 were retrieved for immunohistochemical analysis of EGFR expression.

**Results:**

The data showed that EGFR protein was overexpressed in 187 of 243 (77%) ESCC tissues. Elevated expression was associated with higher pathologic tumor stages (*P =* 0.001), lymph node metastasis (*P =* 0.002), and higher Union for International Cancer Control (UICC) stage (*P* <0.0001), as well as poorer disease-free survival and overall survival of ESCC patients (*P* <0.0001). A multivariate analysis showed that overexpression of EGFR protein was an independent factor for disease-free survival (*P =* 0.003) and overall survival (*P =* 0.001) of these patients. Subgroup analysis of patients with stage IIA (UICC 2002) showed that EGFR overexpression was associated with poorer disease-free survival (*P =* 0.007) and overall survival (*P =* 0.010) of the patients in univariate analyses.

**Conclusions:**

The current study demonstrated that EGFR overexpression was an independent prognostic factor for overall survival and disease-free survival of ESCC patients. However, targeting of EGFR activity using gefitinib or erlotinib could be useful for clinical treatment of ESCC patients.

## Background

Esophageal cancer is a significant worldwide health problem, the sixth most frequent cause of cancer death [1,2]. Surgery remains the primary treatment, but in most cases diagnosis is not determined until after surgery is feasible. Depending on the type of surgery, the five-year survival rate is 15% to 37.8% [3,4]. Studies have shown that prognosis is affected by the number of metastatic lymph nodes [5-7]. Post-surgery pathological studies have confirmed this and have also confirmed an inverse association between survival and the depth of tumor invasion [8,9].

Predictions of post-surgery prognosis are usually based on the Union for International Cancer Control (UICC)/American Joint Committee on Cancer (AJCC) clinical staging system [8,9]. However, determination of tumor stage in esophageal cancer is often imprecise making survival of patients difficult to predict, especially those in the late stages. Thus, there is a need to develop and evaluate biomarkers for predicting survival and treatment response in esophageal cancer.

Elevated levels of epidermal growth factor receptor (EGFR), or increased expression of the *EGFR* gene, have been reported in a number of human cancers of epithelial origin, including head and neck [10], thyroid [11], breast [12], and colon [13,14] cancers. In a subset of these cancers, most notably breast [15], colorectal [13,14], and esophageal cancers [16,17], increased EGFR expression has been associated with advanced disease, tumor metastases, and poor prognosis.

In developed countries, two-thirds of esophageal cancers are adenocarcinoma, but in most of the world, including China, 95% are esophageal squamous cell carcinoma (ESCC) [1,2,18]. In the current retrospective study of cases occurring between 1980 and 1997 at Cancer Hospital, Chinese Academy of Medical Science, we investigated whether EGFR can serve as an independent prognostic factor for overall and disease-free survival of ESCC patients. Associations between EGFR expression in ESCC tissue specimens and patients’ follow-up data were analyzed.

## Methods

### Study population

In this retrospective cohort study, we retrieved the medical records of 243 patients who had undergone esophagectomy for ESCC, without any pre-surgical neoadjuvant or adjuvant chemotherapy or chemoradiotherapy, between 1980 and 1997 at Cancer Hospital, Chinese Academy of Medical Science. These patients had a clinically localized ESCC, including 23, 92, 68, 28, and 32 patients with stage IB, II, IIIA, IIIB, and IIIC disease, respectively, based on the definitions of the UICC 2010 version [9]. Paraffin-embedded tissue specimens were retrieved from the Pathology Department and prepared for construction of tissue microarray and cut into 4 μm-thick sections for immunohistochemistry.

As reported previously [5], for tumor in the upper third of the thoracic segment surgeons performed a three-phase thoraco-abdominal McKeown resection via a right thoracotomy, using the stomach for esophageal replacement. For lesions in the mid and lower third, esophagectomy was performed on the left side using the stomach to establish digestive continuation. In each case, lymph nodes were removed as completely as possible. Juxtatumoral, paraesophageal, superior gastric, left gastric, and paracardial lymph nodes were analyzed individually to determine a final stage classification based on the 2002 and 2010 International Union against Cancer system.

A total of 4,160 lymph nodes (median, 17; range, 0 to 49) were dissected for pathologic staging of the disease after histological examination of hematoxylin-eosin stained tissue sections.

The Institutional Review Board of Cancer Hospital, Chinese Academy of Medical Science approved this study. All patients or their guardians signed an informed consent form to participate in this study. The patients were followed-up regularly every 3 to 6 months after surgery or until death. The last follow-up was in December 2010, which included an esophagograph, chest radiograph, and ultrasound scan of the liver. Treatment failure was defined as any local or distant morphologic evidence of tumor.

For patients with tumor recurrence, treatments included any methods considered useful for relief of suffering. Until the end of follow-up, 77 patients had regional recurrence, and only 35 patients underwent salvage treatment (surgery, 10; radiotherapy, 25). Forty-one patients had organ metastasis; only 12 patients underwent salvage chemotherapy. The median duration of the follow-up time was 25 months (range, 6 to 280 months) after the esophagectomy; the mean duration was 36 months. Five patients were lost at the last follow-up. We counted these as deceased and there was no censure when we calculated overall survival (OS) and disease-free survival (DFS).

### Immunohistochemistry

Tissue microarray sections were deparaffinized in xylene and rehydrated in an ethanol series to water. Antigen retrieval was performed in a citrate buffer (0.01 M, pH 6.0) with a microwave-based method. After incubation with 20% normal serum, the sections were incubated with an anti-EGFR antibody (Novocastra, Cat: #NCL-EGFR-384, Newcastle Upon Tyne, UK) in a humidified chamber overnight at 4°C. The next day, the sections were washed with phosphate buffered saline (PBS) three times, and further incubated using the PV-9000 Polymer Detection System (GBI Labs, cat: #PV-9000, Mukilteo, WA, USA) and color-reacted with 3,3′-diaminobenzidine (DAB) solution (Zhongshan cat# ZLI-9018, Beijing, China) as a chromogen.

**Figure 1:**
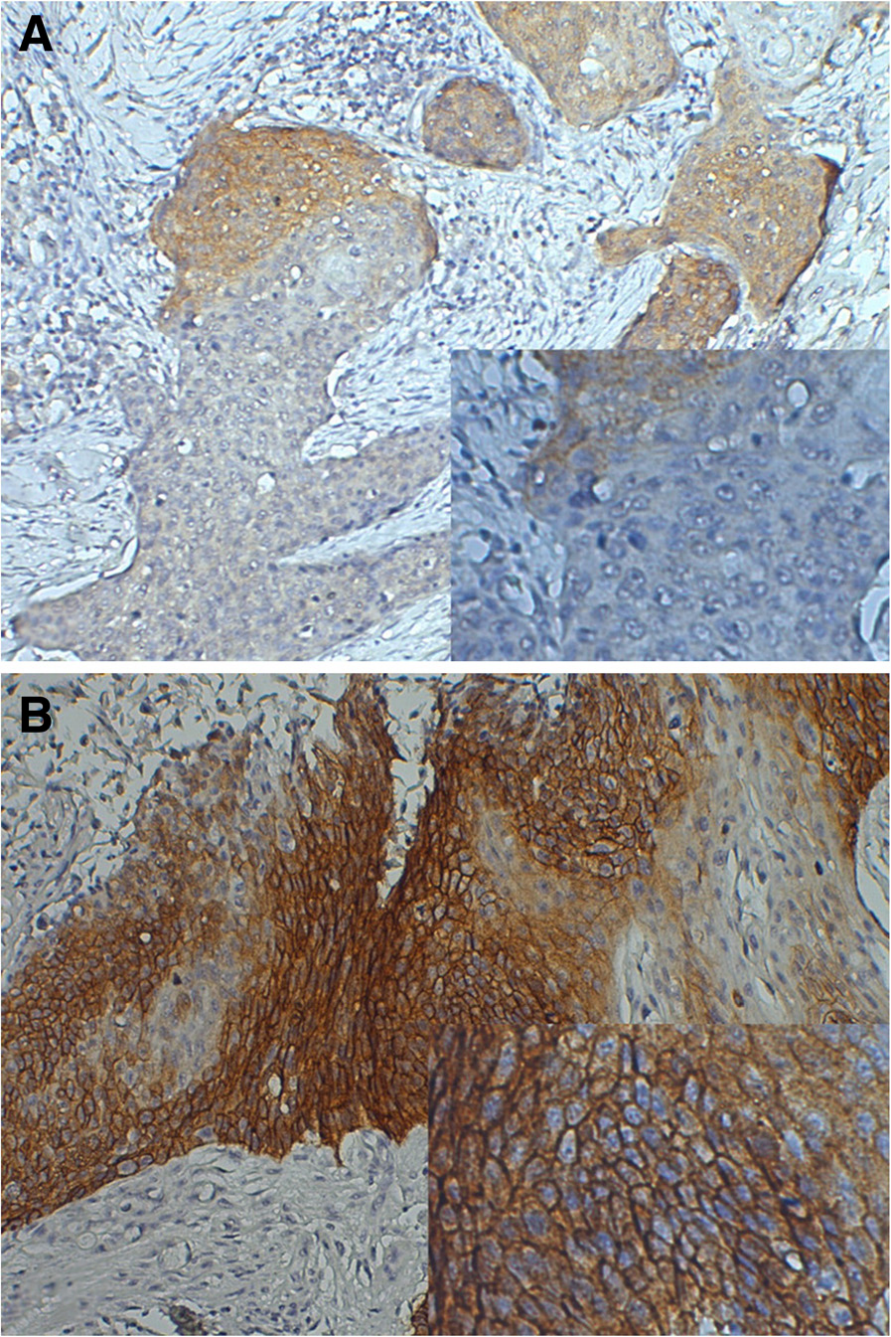
**Immunohistochemical detection of epidermal growth factor receptor (EGFR) protein expression in esophageal squamous cell carcinoma (ESCC) tissues.** Paraffin-embedded tissue sections were processed for immunohistochemical analysis of EGFR expression, which was semiquantitatively scored. **(A)** Low and **(B)** high EGFR expression.

Two experienced pathologists who were blinded to the clinical data reviewed the stained tissue sections. At least five microscopic fields were evaluated. The sections were scored for EGFR expression semiquantitatively based on the color and the percentage of epithelial cells showing membrane staining. The intensity was classified as follows: 0, negative staining; 1, weak staining; 2, moderate staining; 3, strong staining. The percent of positive cells was recorded as: 1, 0 to 25%; 2, 26 to 50%; 3, 51 to 75%; 4, >75%. A final score was achieved by multiplying the intensity (0, 1, 2, or 3) and the percent of positive cells (1, 2, 3, or 4). For data analysis, scores <8 were defined as 'low expression’ (Figure 1A), whereas scores with ≥8 were considered 'high expression’ (Figure 1B).

### Statistical analysis

Categorical data were compared using the chi-squared test. The Kaplan-Meier method was used to construct OS and DFS curves, and the log-rank test was used to determine the statistical significance of differences. DFS was computed from the time of surgery to the time of clinical diagnosis of recurrent tumor, or death without evidence of disease recurrence, at which point the data were censored. OS was calculated from the time of surgery to the time of death from any cause, or to the time of last follow-up, at which point the data were censored. The prognostic significance of clinical and pathologic characteristics was determined using univariate Cox regression analysis. To assess the presence of possible confounding variables, a Cox regression model for multivariate analysis was applied for factors that achieved significance in the univariate analysis. All statistical analyses were performed using SPSS software (version 13.0 for Windows; SPSS, Chicago, IL, USA). Kaplan-Meier survival curves were drawn with GraphPad Prism (version 5.0 for Windows; GraphPad Software, San Diego, CA, USA). A 2-sided significance level of *P* <0.05 was considered statistically significant.

## Results

### Expression of epidermal growth factor receptor protein in esophageal squamous cell carcinoma tissue specimens

We retrieved tissue samples from each of the 243 ESCC patients and immunostained these tissue sections for EGFR expression. Our data showed that in 187 of the 243 cases (77.0%) high levels of EGFR protein were observed in ESCC tissues, while in the remaining cases EGFR levels were nil or low. We then investigated correlations between EGFR expression and clinicopathological data from the patients and found that EGFR expression was associated with advanced tumor stage (*P =* 0.001), tumor lymph node metastasis (*P =* 0.002), and higher pathological stages (UICC 2002 and 2010, *P* <0.001). However, EGFR expression was not associated with age, gender, tumor location, size of the tumor lesion, or histological differentiation (Table 1).

**Table 1:** Patient demographics, clinicopathological features, and epidermal growth factor receptor (EGFR) expression

	**EGFR -Total**		**EGFR- Low**		**EGFR- High**		***P *** **value**^**a**^
	**N**	**%**	**n**	**%**	**N**	**%**	
Gender							0.130
Male	176	77.0	45	80.4	131	70.1	
Female	67	23.0	11	19.6	56	29.9	
Age (y)							0.675
<40	12	4.9	4	7.17	8	4.3	
41 to 50	56	23.0	15	26.8	41	21.9	
51 to 60	101	41.6	21	37.5	80	42.8	
60 to 68	74	30.5	16	28.6	58	31.0	
Length (cm)							0.730
≤5.0	130	53.5	32	57.1	98	52.4	
5.1 to 7.0	90	37.0	20	35.7	70	37.4	
>7.0	23	9.5	4	7.1	19	10.2	
Tumor location							0.137
Upper	33	13.6	5	5.9	28	15.0	
Middle	155	63.8	42	75.0	113	60.4	
Lower	55	22.6	9	16.1	46	24.6	
Histological differentiation							0.607
Well	93	38.3	21	37.5	72	38.5	
Moderate	124	51.0	27	48.2	97	51.9	
Poor	26	10.7	8	14.3	18	9.6	
Tumor invasion							0.001
T2	31	12.8	13	23.2	18	9.6	
T3	168	69.1	41	73.2	127	67.9	
T4	44	18.1	2	3.6	42	22.5	
Lymph node status							0.002
-	130	53.5	40	71.4	90	48.1	
+	113	46.5	16	28.6	97	51.9	
Number of LNM							0.024
1 to 2	65	57.5	8	50.0	57	58.8	
3 to 6	39	34.5	4	25.0	35	36.1	
≥7	9	8.0	4	25.0	5	5.2	
Pathological stage (UICC, sixth edition)							<0.0001
IIA	110	41.3	39	69.6	71	38.0	
IIB	8	5.0	3	5.4	5	2.7	
III	125	51.4	14	25.0	111	59.4	
Pathological stage (UICC, seventh edition)							0.002
IB	23	9.5	10	17.9	13	7.0	
IIA	87	35.8	29	51.8	58	31.0	
IIB	5	2.1	1	1.8	4	2.1	
IIIA	68	28.0	7	12.5	61	32.6	
IIIB	28	11.5	4	7.1	24	12.8	
IIIC	32	13.2	5	8.9	27	14.4	

^a^P, P values were calculated by using chi-square.

### Association of epidermal growth factor receptor expression with overall survival of esophageal squamous cell carcinoma patients

**Figure 2:**
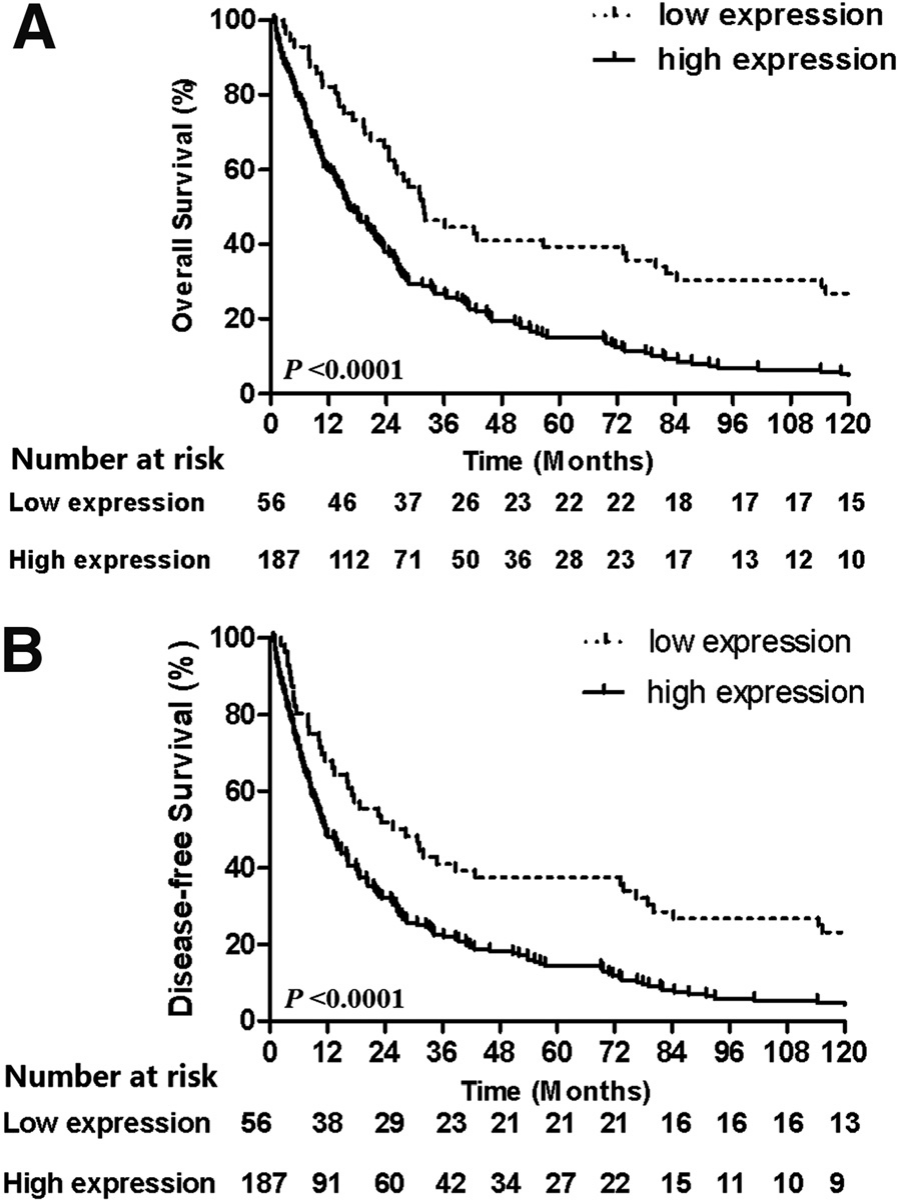
**Association of epidermal growth factor receptor (EGFR) expression with overall and disease-free survival of patients using Kaplan-Meier curves and the log-rank test. (A)** Overall survival and **(B)** disease-free survival.

We investigated correlations between EGFR expression in resected ESCC tissues and survival of ESCC patients and found that the 5-year OS and DFS rates of patients with EGFR expression were 15.0% and 14.4%, respectively, and the median survival times were 16.0 months and 11.6 months. In contrast, the 5-year OS and DFS rates for patients with no or low EGFR expression in ESCC tissues were 39.3% and 37.5%, respectively, and the median survival times were 31.7 and 25.7 months (Figure 2A,B). The differences in OS and DFS between these two groups are statistically significant (*P* < 0.0001, both).

### Univariate and multivariate analysis of overall survival and disease-free survival of esophageal squamous cell carcinoma patients

To identify the important prognostic factors for ESCC, we performed univariate and multivariate analyses. The univariate analysis data showed that tumor size, pathological tumor stage, positive lymph nodes, tumor-node-metastasis (TNM) stage (UICC, sixth or seventh edition), and EGFR expression all affected the OS and DFS of the patients (Table 2). The multivariate analysis further showed that histological grade, positive lymph nodes, tumor size and EGFR expression were independent prognostic factors for OS and DFS of these patients (Table 3).

**Table 2:** Univariate analysis of disease-free survival (DFS) and overall survival (OS) of esophageal squamous cell carcinoma (ESCC) patients

	**N**	**Disease-free survival**			**Overall survival**		
		**HR**	**95% CI**	***P *** **value**	**HR**	**95% CI**	***P *** **value**
Gender							
Male (ref)	176	1			1		
Female	67	1.202	0.894 to 1.616	0.222	1.223	0.906 to 1.650	0.188
Age (y)				0.154			0.178
<40 (ref)	12	1			1		
41 to50	56	0.531	0.265 to 1.066	0.075	0.559	0.278 to 1.121	0.101
51 to 60	101	0.720	0.500 to 1.037	0.078	0.716	0.496 to 1.035	0.075
60 to 68	74	0.857	0.631 to 1.164	0.324	0.875	0.643 to 1.190	0.395
Length (cm)				0.008			0.011
≤5.0 (ref)	130	1			1		
5.1 to 7.0	90	0.546	0.345 to 0.864	0.100	0.563	0.356 to 0.890	0.014
>7.0	23	0.762	0.477 to 1.218	0.256	0.781	0.488 to 1.248	0.301
Tumor location				0.104			0.204
Upper (ref)	33	1			1		
Middle	155	1.611	1.029 to 2.520	0.037	1.502	0.961 to 2.349	0.075
Lower	55	1.280	0.925 to 1.772	0.136	1.166	0.842 to 1.615	0.354
Histological differentiation				0.012			0.325
Well	93	1			1		
Moderate	124	1.418	1.064 to 1.891	0.017	1.296	0.972 to 1.727	0.077
Poor	26	1.271	1.059 to 1.526	0.010	1.247	1.039 to 1.497	0.018
EGFR expression							
Low (ref)	56	1			1		
High	187	1.974	1.413 to 2.758	<0.0001	2.075	1.485 to 2.899	<0.0001
Tumor stage				<0.0001			<0.0001
T2 (ref)	31	1			1		
T3	168	1.303	0.874 to 1.940	0.192	1.320	0.886 to 1.967	0.170
T4	44	1.468	1.146 to 1.880	0.002	1.532	1.194 to 1.965	0.002
N stage^a^							
N0 (ref)	130	1			1		
N1	113	2.203	1.676 to 2.897	<0.001	2.267	1.723 to 2.983	<0.001
Stage^a^				<0.0001			<0.0001
IIA (ref)	110	1			1		
IIB	8	1.343	0.650 to 2.776	0.640	1.253	0.607 to 2.585	0.541
III	125	1.539	1.336 to 1.771	<0.0001	1.583	1.373 to 1.824	<0.0001
N stage^b^				<0.0001			<0.0001
N0 (ref)	130	1			1		
N1	65	1.833	1.339 to 2.509	<0.0001	1.882	1.374 to 2.578	<0.0001
N2	39	1.682	1.384 to 2.044	<0.0001	1.756	1.442 to 2.138	<0.0001
N3	9	1.701	1.338 to 2.161	<0.0001	1.532	1.210 to 1.940	<0.0001
Stage^b^				<0.0001			<0.0001
IB (ref)	23	1			1		
IIA	87	0.969	0.596 to 1.576	0.899	0.939	0.577 to 1.529	0.801
IIB	5	0.971	0.590 to 1.599	0.908	0.908	0.555 to 1.486	0.702
IIIA	68	1.283	1.082 to 1.523	0.003	1.289	1.086 to 1.529	0.003
IIIB	28	1.386	1.168 to 1.644	<0.0001	1.433	1.203 to 1.708	<0.0001
IIIC	32	1.280	1.123 to 1.458	<0.0001	1.306	1.145 to 1.489	0.967

HR, hazard ratio; CI, confidence interval; *P* value was calculated by using a log-rank test; ref, reference.

^a^UICC, sixth edition.

^b^UICC, seventh edition.

**Table 3:** Multivariate Cox regression analysis of disease-free survival (DFS) and overall survival (OS)

	**Overall survival**		**Disease-free survival**	
	**HR (95% CI)**	***P *** **value**	**HR (95% CI)**	***P *** **value**
Histological grade	1.804 (1.282 to 2.540)	0.062	1.301 (1.060 to 1.597)	0.012
Tumor stage	1.191 (0.797 to 1.778)	0.394	1.178 (0.790 to 1.758)	0.422
Lymph node status	2.028 (1.529 to 2.688)	<0.0001	1.888 (1.423 to 2.504)	<0.0001
EGFR expression	1.804 (1.282 to 2.504)	0.001	1.681 (1.192 to 2.369)	0.003
Tumor size (length)	1.331 (1.090 to 1.625)	0.005	1.334 (1.093 to 1.627)	0.005

HR, hazard ratio; CI, confidence interval; EGFR, epidermal growth factor receptor.

### Subgroup analyses of epidermal growth factor receptor expression and overall survival of patients

To further confirm the usefulness of EGFR as a prognostic factor, we compared subgroups of patients who tested either negative or positive for metastasis in lymph nodes (130 and 113 patients, respectively). In the negative lymph node group, the 5-year OS (DFS) rate was 28.9% (24.0%) in patients who expressed EGFR, and 50.0% (47.5%) for patients with low levels or no EGFR expression (*P =* 0.002 (*P =* 0.001)). However, in the 113 cases with positive lymph nodes, the 5-year OS rates were 5.2% for patients with high EGFR expression, and 12.5% for those with low expression (*P =* 0.067).

For the 110 patients with stage T2-3N0M0 IIA disease (2002 UICC), the 5-year OS (DFS) rate was 29.6% (28.0%) for those with high-EGFR expression, and 48.7% (46.7%) for those with low-EGFR expression (*P =* 0.010 (*P =* 0.007)). Furthermore, in 87 cases of patients with stage T3N0M0 IIA (UICC seventh edition) disease, the 5-year OS rate for patients with high EGFR expression was significantly lower than those with low EGFR expression (29.3% compared with 48.3%; *P =* 0.026).

## Discussion

EGFR overexpression has been reported previously in 40 to 84% of esophageal cancer tissues [19-21]. Significant associations have been found between the level of EGFR expression and some clinicopathological characteristics, including tumor differentiation, tumor stage, lymph node status, and UICC TNM stages [19,22,23]. However, whether or not EGFR expression is associated with the survival of esophageal cancer patients remains controversial. Several researchers have shown that EGFR overexpression was associated with poor OS and DFS of esophageal cancer patients [22-24], whereas others have demonstrated that EGFR overexpression had no association with either survival rates [25,26]. These inconsistent conclusions drawn from different studies may be due to an insufficient number of cases or duration of follow-up, or the methods used to detect EGFR expression.

In the present study, we retrospectively retrieved ESCC tissue specimens from 243 patients, which had been collected from 1980 to 1997, and followed these patients for more than 13 years. Moreover, with these specimens we performed standard immunohistochemistry using an anti-EGFR monoclonal antibody from Novocastra, an international company with an excellent reputation for providing immunohistochemistry-quality antibodies, to provide more informative data. We found that EGFR protein was highly expressed in 77% of these tissue specimens. High EGFR expression was associated with tumor invasion, lymph node metastasis, numbers of lymph node metastases, and UICC 2002 and 2010 TNM stages in these esophageal cancer patients. These data are consistent with previous studies [19,22,23]. More importantly, OS and DFS rates were significantly lower in patients with high EGFR expression than in patients with little or no EGFR expression. Further multivariate analysis showed that EGFR expression is an independent prognostic factor for ESCC patients. A future study will investigate how the EGFR signaling pathway contributes to esophageal cancer progression or chemotherapy resistance in ESCC patients.

Until now, pathological features of ESCC such as tumor stage, lymph node status, and tumor distant metastasis have been widely used as prognostic or chemotherapy indicators in addition to more detailed subgroup staging. The latter staging system may better predict prognosis since it is not uncommon that prognosis differs even in patients with the same clinical stage. For instance, the 5-year OS rate of patients with stage IIa disease (according to UICC 2002, T2-3N0M0) after esophagectomy was about 50%, and the remaining patients died within five years because of tumor recurrence or metastasis. Rice *et al*. [7] showed that the 5-year OS rate was about 60% for T2N0M0 esophageal cancer and about 50% for T3N0M0, a difference that is statistically significant. Subsequently, stage IIa disease (UICC 2002), which includes T2N0M0 and T3N0M0, was changed to Ib (T2N0M0) and IIa (T3N0M0) in the UICC 2009 edition.

In the current study the 5-year OS rates of T2N0M0 and T3N0M0 diseases were 39.1% and 35.6%, respectively, a difference that was not statistically significant (*P* = 0.801). However, in the same group of patients, the levels of EGFR expression (that is, high levels compared with low) significantly influenced both the 5-year OS and DFS rates. This suggests that EGFR is a useful biomarker and provides a precise tool for prediction of survival of esophageal cancer patients. Use of EGFR expression could contribute to staging and a more accurate prognostic prediction compared with UICC staging alone, especially for patients with stage T2/T3N0M0. Furthermore, our current multivariate analysis showed that EGFR expression and lymph node status were independent prognostic factors to predict survival of esophageal cancer patients, whereas tumor stage was not able to predict survival. These results suggest that tumor stage alone was not as sensitive as EGFR and lymph node status in predicting the prognosis of patients.

EGFR is the cell-surface receptor for members of the epidermal growth factor family. It is activated by binding to its specific ligands, including epidermal growth factor (EGF) and transforming growth factor α (TGFα). This binding then activates downstream gene pathways such as the mitogen-activated protein kinase (MAPK), protein kinase B (Akt), and c-Jun N-terminal kinase (JNK) pathways, to lead to DNA synthesis and cell proliferation, migration, and invasion [27]. Clinically, *EGFR* mutation and aberrant overexpression may lead to human carcinogenesis and tumor progression, including esophageal cancer [14]. For the past decade, targeting EGFR activity using gefitinib and erlotinib has been used successfully to treat certain lung and colorectal cancer patients [28]. Thus, such treatment may extend to esophageal cancer patients with high EGFR expression and provide a survival benefit.

## Conclusions

In summary, in the present study we found that EGFR expression was associated with tumor invasion, lymph node status, number of lymph node metastases, and UICC TNM staging in ESCC patients. This study also showed that the OS and DFS rates were significantly lower in ESCC patients with high EGFR expression than in those with low EGFR expression. The multivariate analysis indicated that EGFR expression and lymph node metastasis were independent factors for predicting ESCC prognosis. Furthermore, ESCC patients at stage T2-3N0M0 could be considered to have better or poor prognosis based on the EGFR expression level in tissues. Therefore, our current data suggest that EGFR expression should be included as a supplement to UICC staging, especially for T2/T3N0M0 and lymph node-negative patients.

## Abbreviations

DFS: Disease-free survival; EGFR: Epidermal growth factor receptor; ESCC: Esophageal squamous cell carcinoma; OS: Overall survival; TNM: Tumor-node-metastasis; UICC: Union for international cancer control.

## Competing interests

There is no conflict of interest involved in this work.

## Authors’ contributions

HJ and XZ designed the study, with assistance from ZH, ZX, LX, ZW, SK, WL, XN. WQ and ZH analyzed the data. All authors helped to interpret the findings. WQ and XZ wrote the manuscript, which was approved by all authors. All authors read and approved the final manuscript.

## References

[B1] Chen W Zeng H Zheng R Zhang S He J Cancer incidence and mortality in China, 2007 Chin J Cancer Res 2012 11 1 8 23359628 10.1007/s11670-012-0001-6 PMC3555260

[B2] Jemal A Bray F Center MM Ferlay J Ward E Forman D Global cancer statistics CA Cancer J Clin 2011 11 69 90 21296855 10.3322/caac.20107

[B3] Bancewicz J Clark P Smith D Donnelly R Fayers P Weeden S Girling D Hutchinson T Harvey A Lyddiard J Al-Jilaihawi A Bown S Cottier B Jeyasingham K Lea R Matthews H Moghissi K Morritt G Myskow M Pagliero K Rowland C Yosef H Med Res Council Oesophageal Canc Surgical resection with or without preoperative chemotherapy in esophageal cancer: a randomised controlled trial Lancet 2002 11 1727 1733 10.1016/S0140-6736(02)08651-8 12049861

[B4] Kelsen DP Ginsberg R Pajak TF Sheahan DG Gunderson L Mortimer J Estes N Haller DG Ajani J Kocha W Minsky BD Roth JA Chemotherapy followed by surgery compared with surgery alone for localized esophageal cancer N Engl J Med 1998 11 1979 1984 9869669 10.1056/NEJM199812313392704

[B5] Xiao ZF Yang ZY Miao YJ Wang LH Yin WB Gu XZ Zhang DC Sun KL Chen GY He J Influence of number of metastatic lymph nodes on survival of curative resected thoracic esophageal cancer 7.patients and value of radiotherapy: report of 549 cases Int J Radiat Oncol Biol Phys 2005 11 82 90 15850906 10.1016/j.ijrobp.2004.08.046

[B6] Kawahara K Maekawa T Okabayashi K Shiraishi T Yoshinaga Y Yoneda S Hideshima T Shirakusa T The number of lymph node metastases influences survival in esophageal cancer J Surg Oncol 1998 11 160 163 9530885 10.1002/(sici)1096-9098(199803)67:3<160::aid-jso3>3.0.co;2-7

[B7] Rice TW Rusch VW Apperson-Hansen C Allen MS Chen LQ Hunter JG Kesler KA Law S Lerut TE Reed CE Salo JA Scott WJ Swisher SG Watson TJ Blackstone EH Worldwide esophageal cancer collaboration Dis Esophagus 2009 11 1 8 19196264 10.1111/j.1442-2050.2008.00901.x

[B8] Sobin LCW Wittekind C International Union Against Cancer (UICC) TNM Classification of Malignant Tumours 2002 6th New York: John Wiley and Sons

[B9] Sobin LH Gospodarowicz MK Wittekind C International Union Against Cancer (UICC) TNM Classification of Malignant Tumours 2010 7th New York: Wiley-Liss

[B10] Gorski DH Beckett MA Jaskowiak NT Calvin DP Mauceri HJ Salloum RM Seetharam S Koons A Hari DM Kufe DW Weichselbaum RR Blockage of the vascular endothelial growth factor stress response increases the antitumor effects of ionizing radiation Cancer Res 1999 11 3374 3378 10416597

[B11] Kanamori A Abe Y Yajima Y Manabe Y Ito K Epidermal growth factor receptors in plasma membranes of normal and diseased human thyroid glands J Clin Endocrinol Metab 1989 11 899 903 2715291 10.1210/jcem-68-5-899

[B12] Hoos A Urist MJ Stojadinovic A Mastorides S Dudas ME Leung DH Kuo D Brennan MF Lewis JJ Cordon-Cardo C Validation of tissue microarrays for immunohistochemical profiling of cancer specimens using the example of human fibroblastic tumors Am J Pathol 2001 11 1245 1251 11290542 10.1016/S0002-9440(10)64075-8 PMC1891917

[B13] Mayer A Takimoto M Fritz E Schellander G Kofler K Ludwig H The prognostic significance of proliferating cell nuclear antigen, epidermal growth factor receptor, and mdr gene expression in colorectal cancer Cancer 1993 11 2454 2460 8095852 10.1002/1097-0142(19930415)71:8<2454::aid-cncr2820710805>3.0.co;2-2

[B14] Nicholson RI Gee JM Harper ME EGFR and cancer prognosis Eur J Cancer 2001 11 Suppl 4 S9 S15 11597399 10.1016/s0959-8049(01)00231-3

[B15] Perez EA The role of adjuvant monoclonal antibody therapy for breast cancer: rationale and new studies Curr Oncol Rep 2001 11 516 522 11595120 10.1007/s11912-001-0073-9

[B16] Gibault L Metges JP Conan-Charlet V Lozac’h P Robaszkiewicz M Bessaguet C Lagarde N Volant A Diffuse EGFR staining is associated with reduced overall survival in locally advanced oesophageal squamous cell cancer Br J Cancer 2005 11 107 115 15986037 10.1038/sj.bjc.6602625 PMC2361490

[B17] Yano H Shiozaki H Kobayashi K Yano T Tahara H Tamura S Mori T Immunohistologic detection of the epidermal growth factor receptor in human esophageal squamous cell carcinoma Cancer 1991 11 91 98 1702347 10.1002/1097-0142(19910101)67:1<91::aid-cncr2820670118>3.0.co;2-a

[B18] Siegel R Ahmedin Jemal D Cancer Facts & Figures 2013 Atlanta: American Cancer Society, Inc.

[B19] Hanawa M Suzuki S Dobashi Y Yamane T Kono K Enomoto N Ooi A EGFR protein overexpression and gene amplification in squamous cell carcinomas of the esophagus Int J Cancer 2006 11 1173 1180 16161046 10.1002/ijc.21454

[B20] Miyawaki M Hijiya N Tsukamoto Y Nakada C Kawahara K Moriyama M Enhanced phosphorylation of the epidermal growth factor receptor at the site of tyrosine 992 in esophageal carcinomas APMIS 2008 11 1097 1106 19133013 10.1111/j.1600-0463.2008.01125.x

[B21] Boone J van Hillegersberg R Offerhaus GJ van Diest PJ Borel Rinkes IH Ten Kate FJ Targets for molecular therapy in esophageal squamous cell carcinoma: an immunohistochemical analysis Dis Esophagus 2009 11 496 504 19302210 10.1111/j.1442-2050.2009.00951.x

[B22] Wang KL Wu TT Choi IS Wang H Resetkova E Correa AM Hofstetter WL Swisher SG Ajani JA Rashid A Albarracin CT Expression of epidermal growth factor receptor in esophageal and esophagogastric junction adenocarcinomas: association with poor outcome Cancer 2007 11 658 667 17211865 10.1002/cncr.22445

[B23] Wilkinson NW Black JD Roukhadze E Driscoll D Smiley S Hoshi H Geradts J Javle M Brattain M Epidermal growth factor receptor expression correlates with histologic grade in resected esophageal adenocarcinoma J Gastrointest Surg 2004 11 448 453 15120370 10.1016/j.gassur.2004.01.006

[B24] Hoshino M Fukui H Ono Y Sekikawa A Ichikawa K Tomita S Imai Y Imura J Hiraishi H Fujimori T Nuclear expression of phosphorylated EGFR is associated with poor prognosis of patients with esophageal squamous cell carcinoma Pathobiology 2007 11 15 21 17496429 10.1159/000101047

[B25] Gotoh M Takiuchi H Kawabe S Ohta S Kii T Kuwakado S Katsu K Epidermal growth factor receptor is a possible predictor of sensitivity to chemoradiotherapy in the primary lesion of esophageal squamous cell carcinoma Jpn J Clin Oncol 2007 11 652 657 17940077 10.1093/jjco/hym089

[B26] Kii T Takiuchi H Kawabe S Gotoh M Ohta S Tanaka T Kuwakado S Nishitani H Katsu K Evaluation of prognostic factors of esophageal squamous cell carcinoma (stage II-III) after concurrent chemoradiotherapy using biopsy specimens Jpn J Clin Oncol 2007 11 583 589 17709606 10.1093/jjco/hym077

[B27] Oda K Matsuoka Y Funahashi A Kitano H A comprehensive pathway map of epidermal growth factor receptor signaling Mol Syst Biol 2005 11 2005 0010 16729045 10.1038/msb4100014 PMC1681468

[B28] Paez JG Janne PA Lee JC Tracy S Greulich H Gabriel S Herman P Kaye FJ Lindeman N Boggon TJ Naoki K Sasaki H Fujii Y Eck MJ Sellers WR Johnson BE Meyerson M EGFR mutations in lung cancer: correlation with clinical response to gefitinib therapy Science 2004 11 1497 1500 15118125 10.1126/science.1099314

